# From Inner Sense to Outer Action: Interoception and Impulsive Compulsive Behavior Disorders in Parkinson's Disease

**DOI:** 10.1002/mdc3.70187

**Published:** 2025-06-23

**Authors:** Chiara Baiano, Francesca Morgante, Diana Goeta, Chiara Giacobbe, Marianna Amboni, Carmine Vitale, Elena Makovac, Benedetta Demartini, Gabriella Santangelo, Lucia Ricciardi

**Affiliations:** ^1^ Department of Psychology University of Campania Luigi Vanvitelli Caserta Italy; ^2^ Neurosciences and Cell Biology Institute, St George's University of London London UK; ^3^ Department of Medicine, Surgery and Dentistry Centre for Neurodegenerative Diseases (CEMAND), University of Salerno Salerno Italy; ^4^ Department of Medical Human Movement and Well‐being Sciences, University “Parthenope” Naples Italy; ^5^ Clinical and Scientific Institutes (ICS) Maugeri Hermitage Naples Italy; ^6^ Department of Neuroimaging Institute of Psychiatry, Psychology and Neuroscience, King's College London UK; ^7^ Department of Health Sciences University of Milan Milan Italy

**Keywords:** impulse control behaviors, interoception, Parkinson's disease, impulsivity, body signals

## Abstract

**Background:**

Impulsive‐compulsive behavior disorders (ICBDs) in Parkinson's disease (PD) include impulse control disorders (ICDs) and compulsive behaviors, often linked to dopaminergic treatment and altered reward processing. Interoception, the perception of internal bodily signals, plays a key role in emotional regulation and decision‐making. Neuroimaging studies suggest that alterations in interoceptive and reward‐related brain circuits may underlie ICDs in PD. However, the relationship between interoception and ICDs remains underexplored.

**Objective:**

To investigate the relationship between impulse control disorders (ICD) severity and interoceptive abilities—specifically interoceptive accuracy, insight, and self‐reported interoception—in PD patients.

**Methods:**

Fifty‐one participants were assessed using the Questionnaire for Impulsive‐Compulsive Disorders in Parkinson's Disease Rating Scale (QUIP‐RS) alongside measures of interoceptive performance, anxiety, depression, and apathy.

**Results:**

Higher ICD severity predicted increased interoceptive insight and sensibility. No significant link was found between ICD severity and interoceptive accuracy. Additionally, higher Levodopa Equivalent Daily Dose (LEDD) was related to lower interoceptive insight, suggesting a potential modulatory effect of dopaminergic therapy on meta‐cognitive interoception.

**Conclusions:**

These findings highlight the complex interplay between ICDs and interoception, suggesting that heightened self‐reported interoception and interoceptive insight may reinforce impulsive behaviors in PD via enhanced bodily signal salience. This study contributes to understanding the characteristics of ICDs in PD, implicating reward‐related circuits such as the insula and anterior cingulate cortex.

Impulsive‐compulsive behavior disorders (ICBDs) are neuropsychiatric complications associated with Parkinson's disease (PD).^1^ They include impulse control disorders (ICDs), such as pathological gambling, hypersexuality, compulsive shopping, and binge eating, alongside compulsive behaviors like punding and the excessive use of dopamine replacement therapy, also known as dopamine dysregulation syndrome (DDS). ICDs are often conceptualized as behavioral addictions, as they are characterized by repetitive, excessive, and compulsive actions that interfere with daily functioning.^1^ Experimental evidence suggests that ICDs are not a pure drug induced phenomenon, but they are due to the interaction between chronic administration of dopaminergic drugs and disease specific effects on the brain network involved in reward.^2^ For this reason, it has been suggested that in certain predisposed PD patients, dopaminergic drugs administration can be associated with the impaired ability to learn from negative feedback, and encourage impulsive decision‐making.^3^

Interoception refers to the ability of perceiving signals originating from within the body, which provides moment‐by‐moment mapping representing the physiological state of the body.^4^, ^5^ Conscious interoceptive abilities are mainly assessed across three partially dissociable dimensions: (i) interoceptive accuracy, referring to objective performance on tasks such as heartbeat detection or discrimination; (ii) self‐reported interoception, referring to subjective beliefs or confidence in one's interoceptive abilities; and (iii) interoceptive insight, which captures the metacognitive correspondence between actual performance and confidence ratings.^6^, ^7^

Analyzing these multiple levels of processing separately is crucial, as they may reflect distinct underlying processes and can help revealing how different interoceptive dimensions may interact within the same bodily axis.^7^

Interoception plays a critical role in shaping emotional regulation, decision‐making, and self‐awareness.^8^, ^9^ Therefore, interoceptive abilities interact with several cognitive, emotional processing and behaviors in clinical populations and healthy subjects.^5^ Interestingly, dysregulation in interoceptive processing can contribute to the heightened salience of immediate rewards and diminished sensitivity to negative outcomes, mechanisms often observed in addiction and impulsivity‐related conditions.^10^ Recent studies showed a multifaceted relationship between interoception and impulsive behaviors, with interoceptive processes potentially influencing the regulation of impulses through heightened salience of bodily signals.^11^, ^12^, ^13^ For instance, Herman et al^12^, ^13^ emphasize that disrupted interoceptive pathways can exacerbate maladaptive decision‐making by failing to provide accurate internal feedback, linking impulsivity to altered reward anticipation and emotional dysregulation. These findings underscore the role of interoceptive processes in behavioral control and maladaptive tendencies.

Moreover, the insular and anterior cingulate (ACC) cortices—key regions implicated in interoceptive insight—are also central to the reward processing networks.^14^ In PD, previous research has shown that interoceptive abilities are diminished compared to healthy individuals, potentially due to disease‐related neurodegeneration affecting the interoceptive pathways.^15^, ^16^, ^17^

In a systematic review, Martini et al^18^ reported that medicated PD patients with ICDs showed increased metabolism and cerebral blood flow in the insular, orbitofrontal and cingulate cortices. Abnormal ventral‐striatum connectivity with ACC and limbic structures was also reported, suggesting that dysregulation in these circuits may underlie the compulsive and impulsive tendencies in this population.^18^ These findings raise the possibility that alterations in interoceptive processing might reinforce or exacerbate impulsive behaviors, creating a feedback loop that drives the persistence of ICDs.

However, the relationship between interoceptive functioning and ICDs in PD remains underexplored. This study aims to elucidate the relationship between ICDs and interoceptive abilities in PD patients by examining interoceptive accuracy, sensibility, and awareness. We tested possible group differences of patients classified with and without ICDs through means of a validated scale, the Questionnaire for Impulsive‐Compulsive Disorders in PD‐Rating Scales (QUIP‐RS).^1^ Then, we investigated whether ICD severity predicts cardiac interoceptive abilities while accounting for demographic, clinical and neuropsychiatric variables.

## Methods

### Participants

Consecutive PD outpatients referred to the Neurosciences Research Centre of City, St George's University of London, UK and the Parkinson and Movement Disorders Unit of ICS Maugeri Hermitage, Naples, Italy, were screened and enrolled.

To be included in the study, each PD patient had to meet the following criteria: (i) diagnosis of idiopathic PD according to the clinical diagnostic criteria of Movement Disorders Society^19^; (ii) a global cognitive efficiency assessed by means of the Montreal Cognitive Assessment according to published criteria^20^, ^21^, ^22^; (iii) stability on dopaminergic therapy; (iv) absence of any other neurological or psychiatric disorders besides ICD. Considering that PD patients were recruited in two different countries, all clinical and psychometric assessments were administered using instruments validated in both the UK and Italy, ensuring cross‐cultural comparability of the data.^23^

Demographic (ie, gender, age, years of education) and clinical data (ie, disease duration, Levodopa Equivalent Daily Dose, LEDD, severity of motor symptoms assessed by part III of Unified Parkinson's Disease Rating Scale, UPDRS, and stage assessed by Hoehn and Yahr, H&Y) were recorded. All patients were tested in their clinically defined ON medication state. Each PD patient gave their written informed consent to participate in the study, which was approved by the Local Ethics Committees and was performed in accordance with the ethical standards laid down in the 1964 Declaration of Helsinki and its later amendments.

### Assessment of Interoception

Interoceptive accuracy was measured with the heartbeat counting task.^6^, ^7^, ^24^ PD patients were instructed to focus and feel their heartbeats in different time periods (25 s, 35 s and 65 s). PD patients did not receive any cue about the trial duration; and trials were randomized across participants. Before starting the task, a 3‐min baseline was recorded to measure the resting heart rate while the patients were invited to take a comfortable position and rest. Then a 30 s training test was administered. During the execution of the task, PD patients could not use help strategies, such as taking the beating from their wrist, chest or other points. The instructions given to PD patients were adapted from Desmedt et al.^25^ In particular, they were instructed to “feel” the sensation of their heart beating and to report only the number of heartbeats they felt, without guessing. The number of beats provided by each PD patient was then compared with one provided by an ECG trace recorded during the same time. The ECG signal was measured with an electronic device (Polar V800 connected to a gelled H10 belt).^15^ Accuracy of perception was calculated as the mean score of three heartbeat perception intervals according to the following formula: 1/3 ∑ [(1‐(|recorded heartbeats—counted heartbeats|)/recorded heartbeats)].^15^, ^26^ Using this transformation, IA score could vary between 0 and 1, with higher scores indicating smaller differences between recorded and perceived heartbeats (ie, greater accuracy corresponds to higher IA). All trials were manually checked for any missing/ over‐counted heartbeats.

Self‐reported interoception was explored through confidence in the perceived accuracy of performance at the heartbeat counting task: immediately after each trial participants were asked: “how confident are you in your answer?” and had to reply with a number ranging from 0 (“Total guess/No heartbeat awareness”) to 10 (“Complete confidence/Full perception of heartbeat”).^6^, ^7^ Participants did not receive any feedback about their performance.

Metacognitive interoceptive insight was explored performing confidence‐accuracy correlations (ie, Pearson's *r*) during heartbeat counting task.^6^, ^7^

### Assessment of Behavioral Symptoms

The QUIP‐RS^1^, ^23^ was used to evaluate the presence and the severity of ICDs (ie, compulsive gambling, buying, eating, and sexual behavior) and related disorders (ie, medication use, punding, and hobbyism) over the past 4 weeks. For each disorder, the scale consists of four questions rated on a 5‐point Likert scale (ranging from “Never” = 0 to “Very often” = 4) to assess frequently reported thoughts, urges, and desires, difficulties in controlling behaviors, and deceitful behaviors associated with ICBDs. The QUIP‐RS provides scores for each ICD and associated disorder ranging from 0 to 16 (with higher scores indicating greater severity or frequency of symptoms), a total ICD score (score range = 0–64) and a total QUIP‐RS score (score range = 0–112).

The Hamilton Anxiety and Depression Rating Scales (HAM‐A; score range = 0–56^27^; score range = 0–51^28^) and the Apathy Evaluation Scale (AES‐S; score range = 18–72^29^) were used to rate respectively anxiety, depression and apathy.

### Statistical Analyses

Considering clinical diagnosis and the recommended cut‐off scores proposed by Weintraub et al,^1^ we compared PD patients classified as having ICDs and PD patients without ICDs on demographical, clinical, interoceptive, and behavioral variables. A non‐ parametric test (ie, two‐ tailed Mann–Whitney *U* exact test) was used to compare characteristics of the two groups, as the data were not normally distributed.

Spearman correlations were conducted in the entire PD group to explore potential relationships between cardiac interoceptive measures (ie, interoceptive accuracy, self‐reported interoception, and interoceptive insight) and both clinical (ie, disease duration, LEDD, UPDRS‐III, H&Y) and neuropsychiatric characteristics (ie, QUIP‐RS total score; QUIP‐RS ICD score, anxiety, depression, and apathy levels).

Then, linear regressions were conducted to investigate whether cardiac interoceptive abilities (ie, interoceptive accuracy, self‐reported interoception, and interoceptive insight), demographic (ie, sex, age, years of schooling), clinical (ie, MoCA, disease duration, LEDD, UPDRS‐III, H&Y) and neuropsychiatric characteristics (anxiety, depression, and apathy levels) predicted the presence and severity of ICBDs and ICDs.^30^ Specifically, two stepwise linear regression models were carried out, entering dimensions of interoception, demographic, clinical and behavioral features as predictors, and ICBDs and ICDs levels (ie, the QUIP‐RS total score, and the QUIP‐RS ICD score) as separate dependent variables. For all stepwise linear regression models, a mixed method of selection was used. Similarly to the forward method, the predictor with the highest correlation with the outcome variable is entered first, but every time a predictor is added to the model, a removal test is made to constantly reassess the model by removing redundant predictors. Note, that due to normality distribution violations and to control for outliers, we also ran bootstrap regression models with 1000 repetitions and a seed of 1, leading to the same significant results. Statistical analyses were conducted using JASP (version 0.16) and SPSS (version 25) computer software. All reported results are based on two‐tailed p values.

## Results

Our final sample consisted of 51 PD patients (*N* = 31 males), aged 62.03 ± 10.03 years with a mean disease duration of 7.02 ± 3.77. Of these, 19 patients (37.25%) were recruited in Italy and 32 (62.75%) in the UK. Comparisons between Italian and UK PD patients on demographic, clinical, cognitive, and behavioral variables are reported in Supplementary Material S1a.

Among them, 14 PD patients (27%) were classified as having ICDs according to clinical diagnosis and QUIP‐RS validated cut‐offs,^1^ and among these, compulsive eating was observed in 16%, pathological gambling in 4%, hypersexuality in 4%, and compulsive buying in 3%.

Results of the comparison of PD patients with and without ICDs on demographical, clinical, interoceptive, and behavioral variables showed no significant difference between the two groups, except for QUIP‐RS total score and ICD score (Table 1).

**TABLE 1:** Comparisons between PD with and PD without ICD groups on demographic, clinical, cognitive, and behavioral variables

	PD (*N* = 37)	PD‐ICD (*N* = 14)	*U*/*χ* ^2^	*p* (*r*/*V*)
	Mean ± SD	Mean ± SD		
Age (years)	63.73 ± 9.52	57.57 ± 10.32	341	0.08 (0.26)
Education (years)	14.05 ± 3.07	14.43 ± 2.50	233.5	0.58 (0.08)
nM	20	11	2.56	0.11 (0.224)
Disease Duration (years)	7.14 ± 3.56	6.71 ± 4.44	288.5	0.43 (0.11)
UPDRS‐III	13.11 ± 6.23	13.07 ± 8.07	270.5	0.82 (0.03)
H&Y	1.7 ± .54	1.46 ± .52	198.5	0.23 (0.16)
LEDD (mg)	154.73 ± 144.35	174.07 ± 137.29	232	0.57 (0.08)
MoCA	25.99 ± 3.12	27.857 ± 2.03	170	0.06 (0.26)
Interoceptive accuracy	0.35 ± .42	0.54 ± .37	189	0.14 (0.21)
Interoceptive insight	0.01 ± .73	0.16 ± .64	222	0.44 (0.11)
Self‐reported interoception	0.05 ± .02	0.07 ± .02	169.5	0.06 (0.26)
HDRS	7.92 ± 4.17	9.5 ± 5.3	222.5	0.44 (0.11)
HARS	11.13 ± 6.72	15.79 ± 9.97	188.5	0.14 (0.21)
AES	27.49 ± 14.32	34.64 ± 12.56	191	0.15 (0.2)
Total ICD A_D	3.22 ± 3.05	16.57 ± 6.67	0	**<0.001** (0.77)
QUIP Tot A_F	5.58 ± 4.79	25.64 ± 9.85	6.5	**<0.001** (0.74)

*Note*: AES, Apathy Evaluation Scale; H&Y, Hoehn and Yahr Scale; HARS, Hamilton Anxiety Rating Scale; HDRS, Hamilton Depression Rating Scale; ICD, Impulsive Control Beahaviour Disorder; LEDD, Levodopa Equivalent Daily Dose; MoCA, Montreal Cognitive Assessment; *N*, number of participants; nM, number of males; PD, Parkinson's disease; QUIP, Questionnaire for Impulsive‐Compulsive Disorders in Parkinson's Disease‐Rating Scales; r/V, effect size; UPDRS, Unified Parkinson's Disease Rating Scale.

As for the whole PD group, Spearman correlations analyses between the different dimensions of interoception showed a significant correlation between (i) interoceptive accuracy and interoceptive insight (rho = −0.319, *p* = 0.023); (ii) interoceptive accuracy and self‐reported interoception (rho = 0.365, *p* = 0.008). No significant correlation was found between interoceptive insight and self‐reported interoception (rho = −0.130, *p* = 0.362). Moreover, significant correlations were found between (i) interoceptive accuracy and QUIP‐RS total score (rho = 0.361, *p* = 0.009); (ii) interoceptive accuracy and QUIP‐RS ICD score (rho = 0.294, *p* = 0.036); (iii) self‐reported interoception and QUIP‐RS total score (rho = 0.285, *p* = 0.045); (iv) self‐reported interoception and QUIP‐RS ICD score (rho = 0.327, *p* = 0.019); (v) interoceptive insight and QUIP‐RS total score (rho = 0.319, *p* = 0.020); (vi) interoceptive insight and LEDD (rho = −0.571, *p* = <0.001).

**Figure 1:**
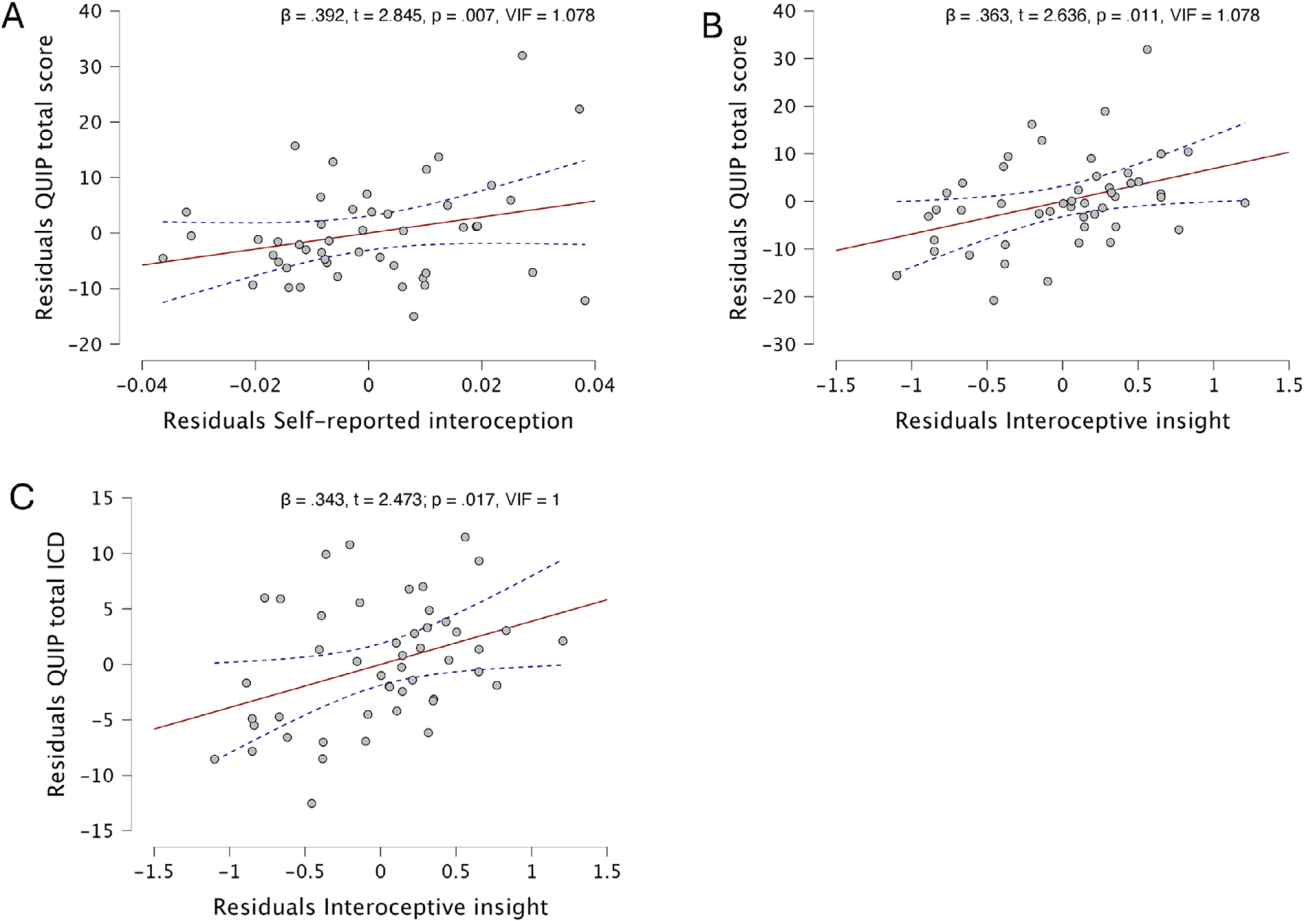
Partial correlation plots of linear regression analyses. (A) Residual plot of QUIP‐RS total score and self‐reported interoception. (B) Residual plot of QUIP‐RS total score and interoceptive insight. (C) Residual plot of QUIP‐RS toal ICD and self‐reported interoception.

Results on the significant linear regressions are reported in Figure 1 and Supplementary material 1b. Data from the stepwise linear regressions showed that the best fitted model for the prediction of QUIP‐RS total score was significant (*F*(2, 49) = 5.931, *p* = 0.005, *R* = .457, *R* ^2^ = 0.209), with predictors being self‐reported interoception (*β* = 0.392, *t* = 2.845, *p* = 0.007, VIF = 1.078) and interoceptive insight (*β* = 0.363, *t* = 2.636, *p* = 0.011, VIF = 1.078). The bootstrap regression model confirmed the same significant result for self‐reported interoception (*B* = 200, SE = 95.8, 95% CI [49.5, 437], *p* = 0.006) and interoceptive insight (*B* = 5.94, SE = 2.108, 95% CI [1.775, 10.087], *p* = 0.004).

The best fitted model for the prediction of QUIP‐RS ICD score was significant (*F*(1, 50) = 6.116, *p* = 0.017, *R* = 0.343, *R* ^2^ = 0.117), with the only predictor self‐reported interoception (*β* = 0.343, *t* = 2.473; p = .017, VIF = 1). The bootstrap regression model confirmed the same significant result (*B* = 107.8, SE = 46.07, 95% CI [34.5, 218], *p* = 0.006).

## Discussion

The present study showed a significant association between ICDs levels and interoceptive processes in PD, specifically regarding interoceptive accuracy and sensibility.

Considering clinical diagnosis and the recommended cut‐off scores, in our study, no significant differences were observed between PD patients with and without ICDs, contrasting with existing literature that highlights higher comorbidity with nonmotor symptoms, including depression and anxiety.^31^ Our findings may reflect sample‐specific characteristics, differences in measurement tools, or variability in dopaminergic medication dosages. However, it is worth noting that PD patients without ICDs in our sample tended to be older and exhibited slightly lower MoCA scores than those with ICDs, although these differences did not reach full statistical significance. This trend aligns with previous findings suggesting that younger age may be a risk factor for developing ICDs in PD, possibly due to increased reward sensitivity or heightened dopaminergic responsivity in younger individuals.^32^, ^33^ Moreover, patients with higher cognitive function may be more prone to engage in goal‐directed or reward‐seeking behaviors, which could facilitate the emergence of impulsive‐compulsive symptoms.^34^

When looking at correlations between interoceptive measures in the whole PD group, we found that higher interoceptive insight was related to lower interoceptive accuracy. The negative correlation suggests a potential dissociation between objective and metacognitive interoceptive abilities. This may indicate a metacognitive misalignment, where PD patients with better actual interoceptive performance underestimate their abilities, whereas those with poorer accuracy may overestimate them. This result further support a multidimensional framework of interoception, reinforcing the value of examining accuracy, insight, and sensibility as distinct yet interacting components in clinical populations.^6^, ^7^, ^8^

Results also showed that higher LEDD correlated with lower interoceptive insight, suggesting that dopaminergic therapy may be related to patients’ meta‐cognitive insight into their interoceptive abilities. This could reflect a “disconnection” within the interoceptive network, possibly due to overstimulation of dopaminergic circuits that modulate self‐awareness.^35^ As interoceptive insight is linked to meta‐cognitive processes in the prefrontal cortex, which can be adversely affected by dopaminergic overstimulation, it is plausible that high doses of dopaminergic medication impair this component by disrupting prefrontal‐limbic connectivity.^36^ On the other hand, a higher LEDD could indicate a more severe disease phenotype associated with reduced metacognition. For instance, previous evidence suggests that patients with PD, particularly in more severe stages, often have diminished awareness of their symptoms.^37^

It is worth noticing that the negative association observed between LEDD and interoceptive insight may partly reflect the impact of dopaminergic therapy on autonomic functions. Dopaminergic medication is indeed known to modulate heart rate and heart rate variability, both of which are directly relevant for interoceptive tasks relying on cardiac signals.^17^, ^38^ However, in our study, all participants were assessed in the ON‐medication state. Future studies adopting within‐subject ON/OFF medication designs, alongside physiological autonomic markers, are warranted to clarify these effects and better characterize how dopaminergic therapy interacts with interoceptive processing in PD.

Results of the multiple linear regressions showed that higher self‐reported interoception and interoceptive insight predicted higher impulsive‐compulsive behaviors assessed by the QUIP‐RS total score. Moreover, higher self‐reported interoception predicted higher ICD levels, assessed by QUIP‐RS ICD score. Generally, one might expect that higher levels of ICDs should be related to lower interoceptive abilities. However, a previous review suggests that higher interoceptive abilities may actually reinforce compulsive behaviors, as seen in addictive disorders.^10^ This relationship has been observed in individuals with both addictive behaviors and impulsivity, suggesting that these individuals are particularly attuned to internal signals that compel them to seek immediate gratification, reinforcing impulsive behaviors.^39^, ^40^ The present study adds to the understanding of this relationship by highlighting a unique pattern in PD patients, where in the interplay between dopaminergic treatment, interoceptive processing, and ICDs suggests a complex interaction that could modulate behavior through enhanced bodily signal perception.

In this regard, we could hypothesize that the increase self‐reported interoception and interoceptive insight stems from alterations in reward‐related brain circuits, particularly in the insula and cingulate cortex, areas known to be implicated in the pathophysiology of ICD.^18^ These brain regions play a critical role in the integration of physiological signals with emotional responses, potentially driving impulsive behavior through increased salience of internal body signals.^40^

However, interestingly, our results did not reveal any significant relationship between interoceptive accuracy and ICBDs. This discrepancy could reflect the complexity of interoceptive pathways also in PD.^6^, ^7^ It is important to note that, although our regression models examined the predictive value of interoceptive abilities on ICBDs, we do not imply a strict causal relationship. Nonetheless, the relationship between interoception and ICBDs is likely to be dynamic and bidirectional. While heightened interoceptive abilities may exacerbate compulsive tendencies, it is equally plausible that sustained engagement in impulsive‐compulsive behaviors could shape bodily awareness through repeated dopaminergic reinforcement and altered insular processing. Future longitudinal investigations are warranted to clarify the directionality of this relationship and its implications for intervention strategies targeting interoceptive insight in this population.

Further limitations of this study should be considered. First, the cross‐sectional design limits causal inferences about the relationship between ICBD severity and interoceptive processes. Longitudinal studies are needed to determine whether changes in interoceptive abilities precede the onset of ICBDs or if these changes are secondary to ICBD development. Second, our sample size was limited, which may reduce the generalisability of the findings. In particular, the relatively small number of patients meeting the clinical threshold for ICDs, may limit generalisability of categorical comparisons. However, our dimensional approach using continuous QUIP‐RS scores helps mitigate this constraint by capturing subthreshold impulsivity features.

Finally, the validity of interoceptive abilities as measured by the heartbeat counting task has been a subject of debate, with concerns raised about confounding factors such as general knowledge of heart rates, as well as the influence of task instructions.^25^, ^41^ To address these limitations and minimize reliance on estimation strategies, we followed Desmedt et al's^25^ protocol, adapting instructions to reduce estimation‐based responses and improve the reliability of participants’ heartbeat counting performance.

In summary, the present study provides a new perspective on the link between cardiac interoceptive abilities and ICDs in PD patients. These insights contribute to the understanding of ICBDs and offer potential avenues for therapeutic strategies targeting interoceptive modulation to manage these behaviors. However, further research is needed to explore these mechanisms and evaluate interventions aimed at balancing interoceptive function in PD patients with ICBDs.

## Author Roles

Research project: 1A. Conception; 1B. Organization; 1C. Execution; Statistical Analysis: 2A. Design; 2B. Execution; 2C. Review and Critique; Manuscript preparation: 3A. Writing of the first draft; 3B. Review and Critique.

C.B.: 1A; 1B; 1C; 2A; 2B; 2C; 3A.

F.M.: 1A; 1B; 2C; 3B.

D.G.: 1B; 1C; 3B.

C.G.: 1C; 2B; 3°.

M.A.: 1°; 2C; 3B.

C. V.: 1°; 2C; 3B.

E.M.: 1A; 1B; 1C.

B.D.: 2C; 3B.

G.S.: 1A; 1B; 2A; 2C; 3B.

L.R.: 1A; 1B; 2A; 2C; 3B.

## Disclosures

**Ethical Compliance Statement:** In the present study, the research was conducted in accordance with ethical standards. Specifically, the study was approved by the Ethical Commitees of the Department of Psychology of the University of Campania, “Luigi Vanvitelli” and of the Neurosciences and Cell Biology Institute, of the St George's University of London. The informed patient consent was obtained in written form at the beginning of each recruitment. We confirm that we have read the Journal's position on ethical publication issues and affirm that this work complies with those guidelines.

**Funding Sources and Conflict of Interest:** CB and GS are supported by #NEXTGENERATIONEU (NGEU) and funded by the Ministry of University and Research (MUR), National Recovery and Resilience Plan (NRRP), project MNESYS (PE0000006)—A Multiscale integrated approach to the study of the nervous system in health and disease (DN. 1553 11.10.2022). CG is supported by #NEXTGENERATIONEU (NGEU) and funded by the Ministry of University and Research (MUR), National Recovery and Resilience Plan (NRRP), project RESTART (Prot. 2022WCMNTT)—Robot Enhanced Social abilities based on Theory of mind for Acceptance of Robot in assistive Treatments (DD no.861, CUP: I53D23003780001). The authors declare that there are no conflicts of interest relevant to this work.

**Financial Disclosures for the previous 12 months:** The authors declare that there are no additional disclosures to report.

## Supporting information


**Supplementary material S1a.** Comparisons between PD patients from UK and from Italy on demographic, clinical, cognitive, and behavioral variables.
**Supplementary material S1b.** Stepwise multiple regression analyses predicting QUIP‐RS total scores and QUIP‐RS ICD scores in PD patients (*N* = 51).

## Data Availability

The data that support the findings of this study are available from the corresponding author upon reasonable request.
