# Supplementary material for: From Inner Sense to Outer Action: Interoception and Impulsive Compulsive Behavior Disorders in Parkinson's Disease
Source: Mov Disord Clin Pract. 2025 Jun 23;12(12):2253–9. doi: 10.1002/mdc3.70187 (PMC12715371; doi:10.1002/mdc3.70187)
Supplement: Supplementary file 1 — Supplementary material S1a. Comparisons between PD patients from UK and from Italy on demographic, clinical, cognitive, and behavioral variables. Supplementary material S1b. Stepwise multiple regression analyses predicting QUIP‐RS total scores and QUIP‐RS ICD scores in PD patients (N = 51). [file MDC3-12-2253-s001.docx]

**Supplementary material 1a.** Comparisons between PD patients from UK and from Italy on demographic, clinical, cognitive, and behavioural variables.

|  | UK PD group (N= 32) | Italian PD group  (N= 19) | U/ χ^2^ | *p (r/V)* |
| --- | --- | --- | --- | --- |
|  | Mean ± SD | Mean ± SD |  |  |
| Age (*ys*) | 64.05 ± 10.63 | 60.84 ± 9.62 | 199.5 | .181 (−0.19) |
| Education (*ys*) | 14.17 ± 1.28 | 13.68 ± 4.36 | 242.5 | .671 (-.06) |
| nM | 21 | 10 | .844 | .266 (.129) |
| Disease Duration (*ys*) | 8.28 ± 4.1 | 4.78 ± 1.43 | 118 | **.001** (.44) |
| UPDRS-III | 15 ± 7 | 9.89 ± 4.8 | 140 | **.007** (-.37) |
| H&Y | 1.4 ± .39 | 1.9 ± .42 | 107 | **<.001** (-.5) |
| LEDD *(mg)* | 141.47 ± 145.62 | 191.32 ± 131.65 | 194.5 | .142 (-.21) |
| MoCA | 27.95 ± 1.94 | 24.05 ± 2.79 | 55 | **<.001** (-.64) |
| Interoceptive accuracy | .50 ± .31 | .24 ± .51 | 207 | .244 (-.17) |
| Interoceptive insight | .090 ± .711 | .012 ± .709 | 212 | .292 (-.15) |
| Self-reported interoception | .058 ± .020 | .054 ± .024 | 255 | .901 (-.02) |
| HDRS | 8.28 ± 4.94 | 8.47 ± 3.79 | 238 | .621 (-.07) |
| HARS | 12.06 ± 8.11 | 13 ± 7.67 | 232.5 | .539 (-.09) |
| AES | 26.5 ± 15.8 | 34.42 ± 8.99 | 223 | .411 (-.12) |
| Total ICD A_D | 8 ± 7.55 | 5.21 ± 4.91 | 209 | .257 (-.16) |
| QUIP Tot A_F | 13.56 ± 12.39 | 7.21 ± 6.98 | 175 | .060 (-.26) |

*Note.* r/V, effect size; PD, Parkinson’s disease; N, Number of participants; ICD, Impulsive Control Beahaviour Disorder; nM, number of males; UPDRS, Unified Parkinson’s Disease Rating Scale; H&Y, Hoehn and Yahr Scale; LEDD, Levodopa Equivalent Daily Dose; MoCA, Montreal Cognitive Assessment; HDRS, Hamilton Depression Rating Scale; HARS, Hamilton Anxiety Rating Scale; AES, Apathy Evaluation Scale; QUIP, Questionnaire for Impulsive-Compulsive Disorders in Parkinson's Disease-Rating Scales.

**Supplementary material 1b.** Stepwise multiple regression analyses predicting QUIP-RS total scores and QUIP-RS ICD scores in PD patients (N=51).

|  | **QUIP-RS total score** | | | **QUIP-RS ICD score** | | |
| --- | --- | --- | --- | --- | --- | --- |
| **Predictor** | β | t | p | β | t | p |
| **M1** |  |  |  |  |  |  |
| Self-reported interoception | .294 | 2.086 | .043 | .343 | 2.473 | .017 |
| Fit model | F(1,50) = 4.349, p = .043, R = .294, R^2^ = .086 | | | F(1,50) = 6.116, p = .017, R = .343, R^2^ = .117 | | |
| **M2** |  |  |  |  |  |  |
| Self-reported interoception | .392 | 2.845 | .007 | - | - | - |
| Interoceptive insight | .363 | 2.636 | .011 | - | - | - |
| Fit model | F(2, 49) = 5.931, p = .005, R = .457, R^2^ = .209 | | | - | - | - |

*Note.* PD, Parkinson’s disease; N, Number of participants; ICD, Impulsive Control Beahaviour Disorder; QUIP, Questionnaire for Impulsive-Compulsive Disorders in Parkinson's Disease-Rating Scales.
